# Supplementary material for: The Novel Genetic Background of Infectious Bursal Disease Virus Strains Emerging from the Action of Positive Selection
Source: Viruses. 2021 Mar 2;13(3):396. doi: 10.3390/v13030396 (PMC7998436; doi:10.3390/v13030396)
Supplement: Supplementary file 1 [file viruses-13-00396-s001.pdf]

Figure S1. Recombination analysis of the nucleotide sequences of Polish IBDV strain 75/01-Poland-2001. For the recombination analysis the software package RDP v4.97 was used. For simplification purposes only the Bootscan analysis is shown as a method detecting the recombination event. A) Visualization of the recombination event detected by Bootscan analysis, the recombinant strain is denoted and the major parent-recombinant the minor parent-recombinant were denoted in blue and red, respectively. B) Neighbour Joining phylogenetic tree showing the topology organization using the minor parental segment (left) and the major parental segment (right). Minor parental strain is denoted in blue, major parental strain is denoted in green and the recombinant strains denoted by an arrow and highlighted in red.

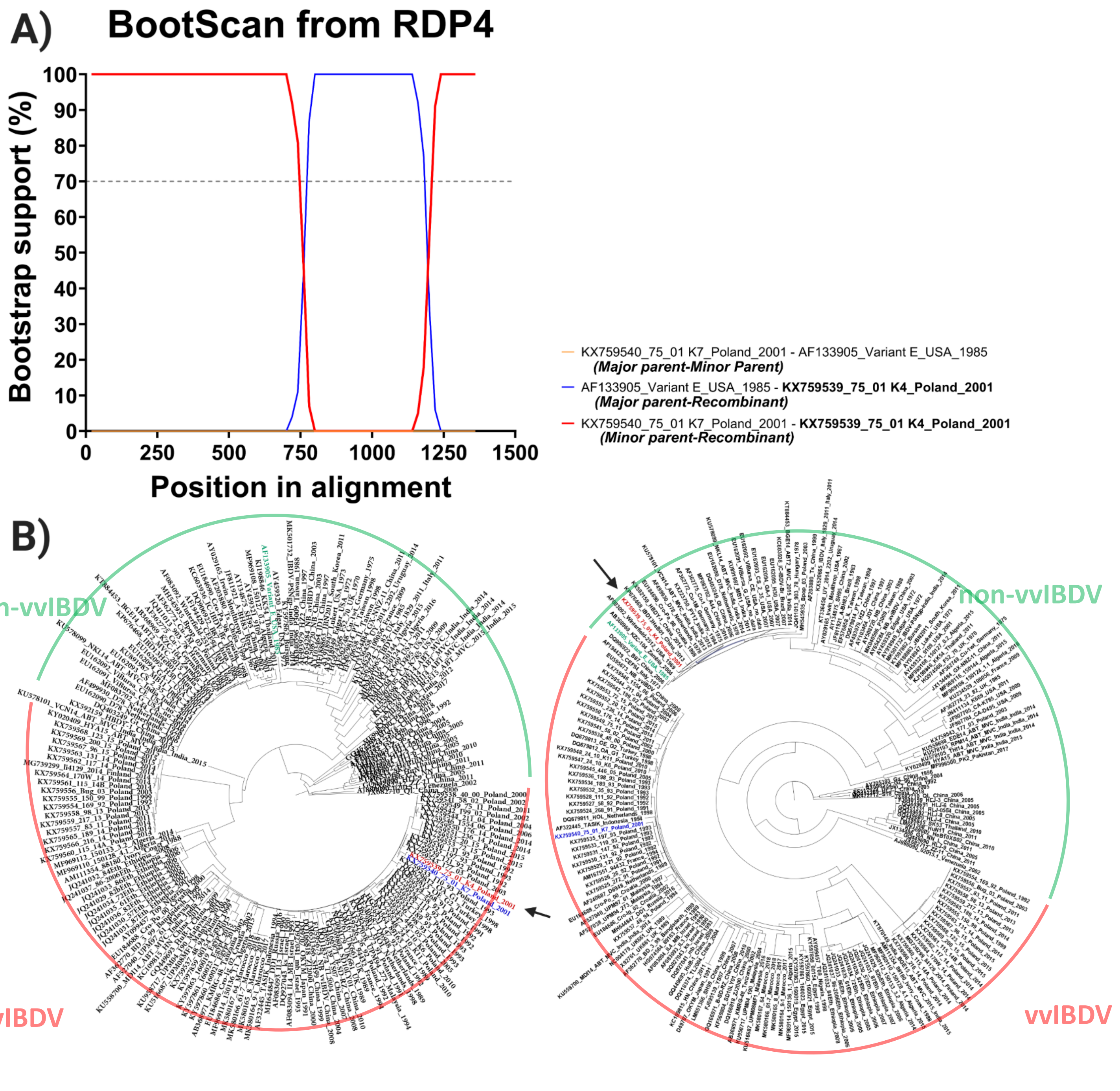

Figure S2. Sequence alignment of ORF2 of Segment A of IBDV. Sequences from the strains used in the experimental infection were used, the strain D78 representative of the attenuate IBDV strain was used for comparison purposes, the location of coding regions for VP2, VP4 and VP3 immature proteins are denoted in rectangle colours (red:VP2, green:VP4 and yellow:VP3), mature protein sequences were denoted in dark coloured rectangle (red:VP2, green:VP4 and yellow:VP3).

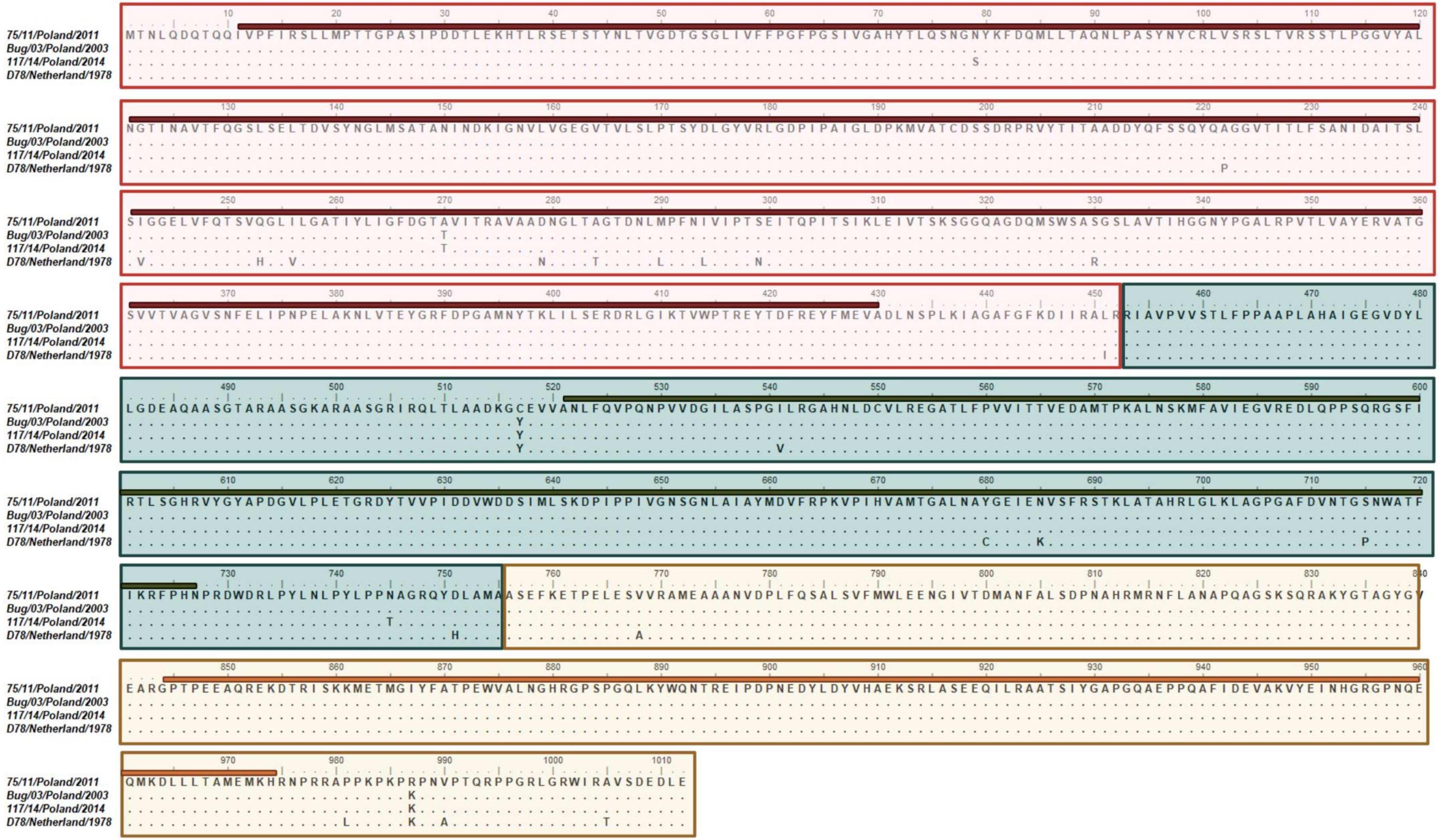

Table S1. PAML branch-site model A analysis to identify branches under episodic positive selection in Polish IBDV strains.

**Foreground branches**	**Parameters$^{n.m}$**	**-lnL$^{n.m}$**	**Parameters$^{a.m}$**	**-lnL$^{a.m}$**	**-2ΔlnL**	**Positively selected sites**
	P_0= 0.0191		P_0= 0.0000			
	P_1= 0.9809		P_1= 0.0000			
	P_{2a}= 0.0000		P_{2a}= 0.9998			
VP2 Clade A1	P_{2b}= 0.0000	2512.644628	P_{2b}= 0.0002	2506.148819	**12.5794****	**206, 207, 210, 215, 221, 222, 230, 234, 237, 246, 254, 261, 282, 286, 302, 304, 312**
	ω_0= 0.0000		ω_0= 1.0000			
	ω_1= 1.0000		ω_1=1.0000			
	ω_2=1.0000		ω_2=**217.4730**			
	P_0= 0.5245		P_0= 0.0000			
	P_1= 0.2737		P_1= 0.0000			
	P_{2a}= 0.1326		P_{2a}= 0.7266			
VP2 Clade A2	P_{2b}= 0.0692	2512.850945	P_{2b}= 0.2734	2518.690369	**11.678848****	0
	ω_0= 1.0000		ω_0= 1.0000			
	ω_1= 1.0000		ω_1= 1.0000			
	ω_2= 1.000		ω_2= **1.6607**			
	P_0= 0.8814		P_0= 0.8730			
	P_1= 0.0311		P_1= 0.1190			
	P_{2a}= 0.0846		P_{2a}= 0.0001			
VP2 Clade A3	P_{2b}= 0.0030	2512.850753	P_{2b}= 0.0001	2518.728187	**11.754868****	0
	ω_0= 1.0000		ω_0= 0.0000			
	ω_1= 1.0000		ω_1= 1.0000			
	ω_2= 1.0000		ω_2= **216.4132**			
	P_0= 0.9580		P_0= 0.9581			
	P_1= 0.0178		P_1= 0.0177			
	P_{2a}= 0.0238		P_{2a}= 0.0238			
VP1 non-vvIBDV	P_{2b}= 0.0004	13348.604162	P_{2b}= 0.0004	13348.60416	0.00004	0
	ω_0= 0.0382		ω_0= 0.0352			
	ω_1= 1.0000		ω_1=1.0000			
	ω_2= 1.0000		ω_2= 1.0000			
	P_0= 0.9745		P_0= 0.9745			
	P_1= 0.0168		P_1= 0.0168			
	P_{2a}= 0.0086		P_{2a}= 0.0086			
VP1 vvIBDV	P_{2b}= 0.0002	13332.88523	P_{2b}= 0.0002	13340.3787	14.98694	0
	ω_0= 0.0375		ω_0= 0.0375			
	ω_1= 1.0000		ω_1= 1.0000			
	ω_2= 1.0000		ω_2= 1.0000			
	P_0= 0.9795		P_0= 0.9795			
	P_1= 0.0105		P_1= 0.0105			
	P_{2a}= 0.0063		P_{2a}= 0.0063			
VP1 transIBDV	P_{2b}= 0.0037	13348.604162	P_{2b}= 0.0037	13338.8682	**19.471922****	**396, 466**
	ω_0= 0.0382		ω_0= 0.0382			
	ω_1= 1.0000		ω_1= 1.0000			
	ω_2= 1.0000		**ω_2= 16.3423**			

lnL: log-likelihood scores; n.m: null model; a.m: alternative model; *p<0.05, χ^2= 3.84; **p<0.01, χ^2= 5.99.

Table S2. Positively selected sites and parameters estimated by the CODEML program implemented in the PAML package.

Gene	Model	log-likelihood score						Positive site
**VP2**	**M1**	-10080.290	p0=0.93345	p1=0.06655				
	**M2**	-10068.144	ω=2.00672	p0=0.93380	p1=0.06399	p2=0.00222		217, 248, 249, 315
	**M7**	-10079.394	p=0.27465	q=1.97108				
	**M8**	-10061.359	ω=1.29072	p0=0.97370	p=0.46792	q=5.09210	p1=0.02630	217, 248, 249, 315

codons selected with a posterior probability greater than 0.99 belonging to the positively selected class ($\omega > 1$).

Table S3. Statistical analysis to avoid false estimations of sites under positive pressure selection.

Gene	Models compared	$-2\Delta l$	d.f	dN/dS
VP2	M1 vs. M2	24.29**	2	2.01
	M7 vs. M8	36.07**	2	1.29

Neutral models (M1 and M7) were compared to selection models (M2 and M8); *, $P < 0.05$ **, $P < 0.01$ ($\chi^2_{0.05,2}$= 5.99, $\chi^2_{0.01,2}$= 9.21). Δl: likelihood-ratio statistic; d.f., degrees of freedom between nested models.

Table S4. Maximum Likelihood estimates of the coefficient of Type-II functional divergence (θ_{II}) from pairwise comparison among VP1 IBDV Polish lineages.

IBDV Polish lineage VP1	θ_{II}ML	θ_{II}SE	θLTR	Qk	p
vvIBDV/transIBDV	0.00744	0.00887	3.15581	115, 116, 117, 189, 212	**p<0.01**
